# Low incidence of new biochemical and clinical hypogonadism following hypofractionated stereotactic body radiation therapy (SBRT) monotherapy for low- to intermediate-risk prostate cancer

**DOI:** 10.1186/1756-8722-4-12

**Published:** 2011-03-27

**Authors:** Eric K Oermann, Simeng Suy, Heather N Hanscom, Joy S Kim, Sue Lei, Xia Yu, Guowei Zhang, Brook Ennis, JoyAnn P Rohan, Nathaniel Piel, Benjamin A Sherer, Devin Borum, Viola J Chen, Gerald P Batipps, Nicholas L Constantinople, Stephen W Dejter, Gaurav Bandi, John Pahira, Kevin G McGeagh, Lucile Adams-Campbell, Reena Jha, Nancy A Dawson, Brian T Collins, Anatoly Dritschilo, John H Lynch, Sean P Collins

**Affiliations:** 1 Department of Radiation Medicine, Georgetown University Hospital, Washington, D.C., USA; 2 Department of Urology, Georgetown University Hospital, Washington, D.C., USA; 3 Department of Radiology, Georgetown University Hospital, Washington, D.C., USA; 4 Department of Oncology, Lombardi Comprehensive Cancer Center, Georgetown University Medical Center, Washington, D.C., USA

## Abstract

**Background:**

The CyberKnife is an appealing delivery system for hypofractionated stereotactic body radiation therapy (SBRT) because of its ability to deliver highly conformal radiation therapy to moving targets. This conformity is achieved via 100s of non-coplanar radiation beams, which could potentially increase transitory testicular irradiation and result in post-therapy hypogonadism. We report on our early experience with CyberKnife SBRT for low- to intermediate-risk prostate cancer patients and assess the rate of inducing biochemical and clinical hypogonadism.

**Methods:**

Twenty-six patients were treated with hypofractionated SBRT to a dose of 36.25 Gy in 5 fractions. All patients had histologically confirmed low- to intermediate-risk prostate adenocarcinoma (clinical stage ≤ T2b, Gleason score ≤ 7, PSA ≤ 20 ng/ml). PSA and total testosterone levels were obtained pre-treatment, 1 month post-treatment and every 3 months thereafter, for 1 year. Biochemical hypogonadism was defined as a total serum testosterone level below 8 nmol/L. Urinary and gastrointestinal toxicity was assessed using Common Toxicity Criteria v3; quality of life was assessed using the American Urological Association Symptom Score, Sexual Health Inventory for Men and Expanded Prostate Cancer Index Composite questionnaires.

**Results:**

All 26 patients completed the treatment with a median 15 months (range, 13-19 months) follow-up. Median pre-treatment PSA was 5.75 ng/ml (range, 2.3-10.3 ng/ml), and a decrease to a median of 0.7 ng/ml (range, 0.2-1.8 ng/ml) was observed by one year post-treatment. The median pre-treatment total serum testosterone level was 13.81 nmol/L (range, 5.55 - 39.87 nmol/L). Post-treatment testosterone levels slowly decreased with the median value at one year follow-up of 10.53 nmol/L, significantly lower than the pre-treatment value (*p *< 0.013). The median absolute fall was 3.28 nmol/L and the median percent fall was 23.75%. There was no increase in biochemical hypogonadism at one year post-treatment. Average EPIC sexual and hormonal scores were not significantly changed by one year post-treatment.

**Conclusions:**

Hypofractionated SBRT offers the radiobiological benefit of a large fraction size and is well-tolerated by men with low- to intermediate-risk prostate cancer. Early results are encouraging with an excellent biochemical response. The rate of new biochemical and clinical hypogonadism was low one year after treatment.

## Background

Recent analyses of clinical data suggest that large radiation fraction sizes are radiobiologically favorable compared to smaller fraction sizes in prostate cancer radiotherapy [1]. The CyberKnife (Accuray, Inc., Sunnyvale, CA) is an FDA-approved radiosurgical device that is ideal for accurately delivering hypofractionated stereotactic body radiation therapy (SBRT) [2]. Treatment is delivered by a linear accelerator mounted on a flexible robotic arm. A few hundred treatment beams are selected from a repertoire of greater than one thousand possible beam directions using inverse treatment planning. These beams are delivered in a non-isocentric, non-coplanar manner via circular collimators of varying sizes. Access to a large number of potential beam trajectories allows delivery of a highly conformal dose with steep dose gradients [3,4]. Unlike standard radiation therapy delivery systems, the CyberKnife system incorporates a dynamic tracking system consisting of an orthogonal pair of diagnostic-quality x-ray imaging devices and software that can locate fiducials implanted within the prostate [5]. This provides updated position information in six dimensions (three translations combined with roll, pitch and yaw rotations) [6] to the robot, which adjusts the targeting of the therapeutic beam during treatment to correct for intra-fraction motion. These features allow for a reduction in the planning target volume (PTV) and potentially the dose to surrounding critical organs. These technical improvements should allow for dose escalation within the prostate while maintaining normal tissue tolerance.

The early efficacy and safety of CyberKnife hypofractionated dose-escalated SBRT have been documented for localized treatment of prostate cancer [7-9]. Stanford's phase II protocol delivered 36.25 Gy in 5 fractions of 7.25 Gy. This dose and fractionation were selected for radiobiologic dose escalation while keeping a constant predicted normal tissue late effect. In King et al.'s report on 41 "low-risk" patients, at a median of 33 months after treatment, the mean PSA was 0.44 ng/ml [7], suggesting a high rate of long-term control [10]. No patient experienced grade 4 toxicity, and only two patients experienced grade 3 late urinary morbidity. Similar results with similar regimens have been reported by others [8,9].

Due to anatomic proximity, the testes are at risk for exposure to scattered radiation during prostate treatment. It has been suggested that the non-coplanar nature of CyberKnife SBRT may increase the risk of testicular irradiation during treatment [11]. The resulting decline in testosterone levels [12,13] could be responsible for the low PSA nadirs [14] obtained with CyberKnife SBRT. If so, the post-treatment PSA response may not accurately reflect the likelihood of long-term tumor control with such treatment [10]. Equally important, the resulting endocrine changes may contribute to post-radiation hypogonadism with subsequent depression, cognitive decline, decreased libido and impotence [15]. Knowledge of the relative risks of hypogonadism due to available treatment options for prostate cancer could affect patients' treatment decisions. In this paper, we report on the use of CyberKnife SBRT as monotherapy for the treatment of 26 prostate cancer patients and show that the risk of new biochemical and clinical hypogonadism is low within the first year after treatment.

## Methods

### Patient Selection

Patients eligible for inclusion in this study had histologically-confirmed low- to intermediate-risk adenocarcinoma of the prostate (clinical stage ≤ T2b, Gleason score ≤ 7, PSA ≤ 20 ng/ml). Exclusion criteria included androgen deprivation therapy, clinically involved lymph nodes on imaging, distant metastases on bone scan, prior pelvic radiotherapy or prior radical prostate surgery. Institutional IRB approval was obtained for this retrospective review.

### SBRT Treatment Planning and Delivery

Four gold fiducials were placed into the prostate prior to treatment planning: two at the apex and two at the base. To allow for fiducial stabilization, planning imaging was performed at least 7 days after fiducial placement. Patients underwent 1.5 T MR imaging followed shortly thereafter by a thin-cut (1.25 mm) CT scan. Both scans were performed with an empty bladder. Patients were advised to adhere to a low-fiber diet, starting at least five days prior to all treatment planning imaging and treatment delivery. They were restricted to nothing by mouth (NPO) the night before, and an enema was administered 1-2 hours prior to imaging and treatment.

**Figure 1:**
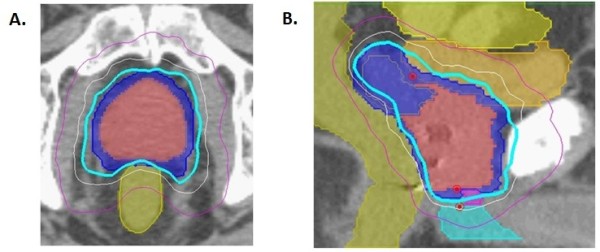
**Treatment planning axial (A) and sagittal (B) computed tomography images demonstrating the GTV (red), CTV and PTV expansion (dark blue), bladder (orange), rectum (green), bowel (yellow), membranous urethra (pink) and penile bulb (light blue)**. Isodose lines shown as follows: Blue 79% (prescription), white 70% and purple 50%.

Fused CT and MR images were used for treatment planning (Figure 1). The gross target volume (GTV) was the prostate. The clinical target volume (CTV) included the prostate and the proximal seminal vesicles to the point where the left and right seminal vesicles separate. The PTV equaled the CTV expanded 3 mm posteriorly and 5 mm in all other dimensions. The prescription dose was 36.25 Gy to the PTV delivered in five fractions of 7.25 Gy over two weeks. The volume of the PTV receiving 36.25 Gy was at least 95%. The prescription isodose line was limited to ≥ 75%, which limited the maximum prostatic urethra dose to 133% of the prescription dose. The rectum, bladder, testes, penile bulb and membranous urethra were contoured structures and evaluated with dose-volume histogram analysis during treatment planning using Multiplan (Accuray Inc., Sunnyvale, CA) inverse treatment planning. Rectal volume receiving 36 Gy was limited to < 1 cc. The rectal dose-volume histogram (DVH) goals were < 50% rectal volume receiving 50% of the prescribed dose, < 20% receiving 80% of the dose, < 10% receiving 90% of the dose, and < 5% receiving 100% of the dose [7]. The empty bladder volume receiving 37 Gy was limited to < 10 cc [8]. Care was taken to avoid treatment beams that directly traversed the testes, and the scatter dose was kept to a minimum. Image-guidance was employed to minimize the required PTV treatment margins. Using computed tomography planning, target volume locations were related to the gold fiducial markers. Position verification was validated several times per minute during treatment using paired, orthogonal, and x-ray images.

### Follow-up

PSA and total testosterone levels were obtained before treatment, one month after the completion of radiation, and during routine follow-up visits every 3 months for the first year. Samples were obtained in the morning and early afternoon to limit the effects of circadian variation [16]. Biochemical hypogonadism was defined as total serum testosterone level below 8 nmol/L [17]. Toxicity was assessed pre-treatment and at 1, 3, 6, 9 and 12 months post-treatment using the National Cancer Institute (NCI) Common Toxicity Criteria (CTC) version 3.0 [18] and the American Urological Association (AUA) symptom score (also known as International Prostate Symptom Score) [19]. Quality of life (QoL) was assessed pre-treatment and at follow-up visits using the Short Form-12 Health Survey (SF-12), the Expanded Prostate Cancer Index Composite (EPIC) [20] and the Sexual Health Inventory for Men (SHIM) [21].

### Statistical Analysis

Skewed continuous variables, e.g., testosterone and PSA, were described as the sample median and range. Categorical variables were described as frequency and percentage. Obtaining PSA, total testosterone, and quality of life measurements sequentially in each patient constitutes a natural control for potentially wide baseline variation across patients. Therefore responses to radiotherapy were assessed using non-parametric pairwise Wilcoxon rank-sum testing [22].

## Results

From January 2009 to June 2009, 26 prostate cancer patients were treated per our institutional protocol. Their median age was 69 years (range, 48-79 years). Similar numbers of Caucasians and African-Americans were enrolled reflecting the distribution of our patient population. Fourteen patients were low-risk, and 12 patients were intermediate-risk per the D'Amico Risk Classification [23]. Table 1 provides detailed patient characteristics.

**Table 1:** Pre-treatment patient characteristics

#	Age	Race	PSA (ng/mL)	T Stage	Gleason Score	Risk Group	Prostate Volume (cc)	AUA	SHIM
1	60	Cau	4.7	1c	3+3	Low	53	3	20

2	69	Cau	6.8	1c	3+4	Intermediate	46	3	14

3	69	Cau	6.1	1c	3+3	Low	29	9	1

4	60	Cau	4.5	1c	3+3	Low	21	3	18

5	71	AA	4.0	1c	2+3	Low	31	16	19

6	72	Cau	5.6	1c	3+3	Low	41	4	1

7	56	AA	5.7	1c	3+3	Low	43	9	16

8	70	Cau	4.9	1c	3+3	Low	23	4	21

9	74	Cau	4.9	1c	3+3	Low	45	10	15

10	78	Cau	8.1	2b	3+3	Intermediate	33	1	3

11	71	Cau	4.9	1c	3+3	Low	33	5	20

12	58	AA	7.9	1c	3+4	Intermediate	37	12	21

13	66	Cau	10.3	1c	3+3	Intermediate	34	14	25

14	74	AA	6.3	1c	4+3	Intermediate	55	9	4

15	70	Cau	6.8	1c	3+3	Low	30	21	20

16	62	Cau	4.0	1c	3+4	Intermediate	30	1	25

17	79	Cau	2.3	2b	3+4	Intermediate	52	5	3

18	48	AA	6.8	1c	3+3	Low	18	8	24

19	73	Cau	6.9	1c	3+4	Intermediate	40	3	4

20	62	Cau	5.6	1c	3+3	Low	25	6	23

21	63	AA	6.2	1c	3+4	Intermediate	42	4	15

22	69	AA	5.8	1c	3+4	Intermediate	42	6	18

23	71	AA	5.9	1c	3+3	Low	34	2	24

24	65	Cau	7.4	1c	4+3	Intermediate	33	7	24

25	78	AA	4.2	2b	4+3	Intermediate	37	10	1

26	67	Cau	4	2a	3+3	Low	49	5	20

**Abbreviations: **Cau - Caucasian; AA - African American; AUA - American Urology Association; SHIM - sexual health inventory for men.

**Figure 2:**
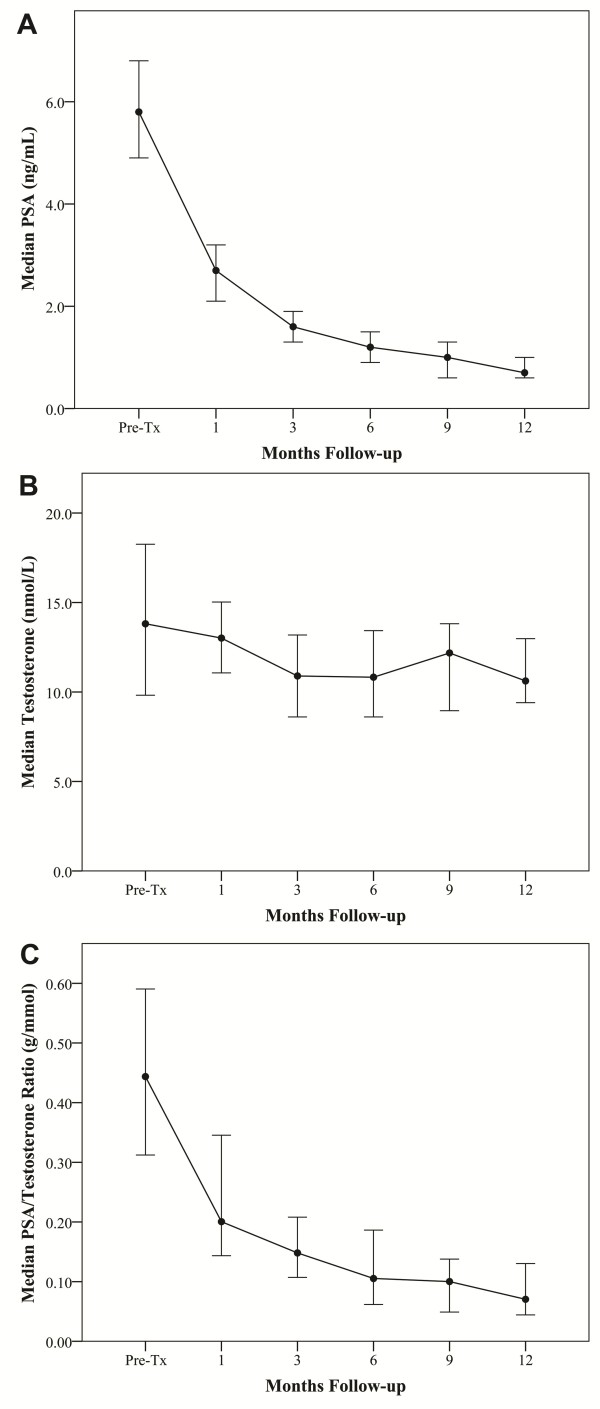
**Pre- and post-treatment (A) PSA levels, (B) total testosterone levels, and (C) PSA/testosterone ratios for all patients**. Error bars indicate 95% confidence intervals.

At a median follow-up of 15 months (range, 13-19 months), the initial PSA response has been favorable, with decreased PSA levels in all patients. The median pre-treatment PSA was 5.75 ng/ml (range, 2.3-10.3 ng/ml); it decreased to a median of 0.7 ng/ml (range, 0.2-1.8 ng/ml) by one year post-treatment (Figure 2A), suggesting a high rate of long term disease control using this treatment regimen [24].

**Figure 3:**
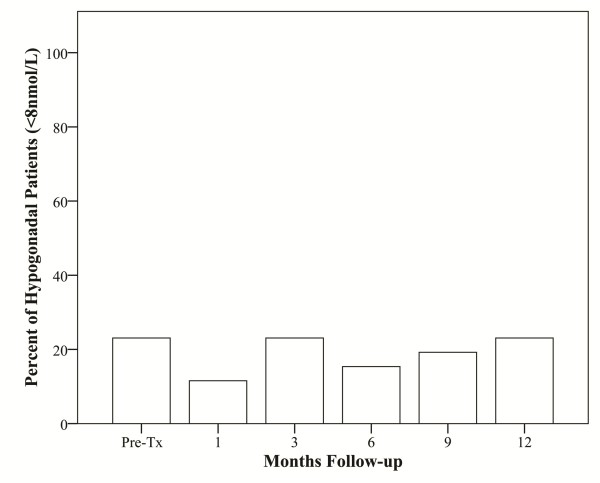
**Comparison of pre-treatment biochemical hypogonadism rates to those at up to 1 year following treatment**.

Consistent with our elderly patient population, pre-treatment total serum testosterone levels were low, ranging from 5.55 nmol/L to 39.87 nmol/L with a median value of 13.81 nmol/L[25]. The median testicular scatter dose was 2.1 Gy (range, 1.1-5.8 Gy). Post-treatment total serum testosterone levels fell in 18 patients (69%) and increased in 8 patients (31%). At one year the median serum testosterone value of 10.53 nmol/L (range, 5.79-22.38 nmol/L) was significantly lower than the pre-treatment value (*p *< 0.013) (Figure 2B). The median absolute fall was small (3.28 nmol/L) and the median percent fall was 23.75%. Pre- and post-treatment median total testosterone levels are shown in Figure 2B. In contrast to the total serum testosterone levels, the PSA to testosterone ratio decreased in all the patients, suggesting that the PSA decrease was not due solely to the drop in testosterone (Figure 2C). Based on the International Society for the Study of the Aging Male (ISSAM) definition (< 8 nmol/L) [18], the pre-treatment and 1-year biochemical hypogonadism rates were identical (Figure 3).

**Figure 4:**
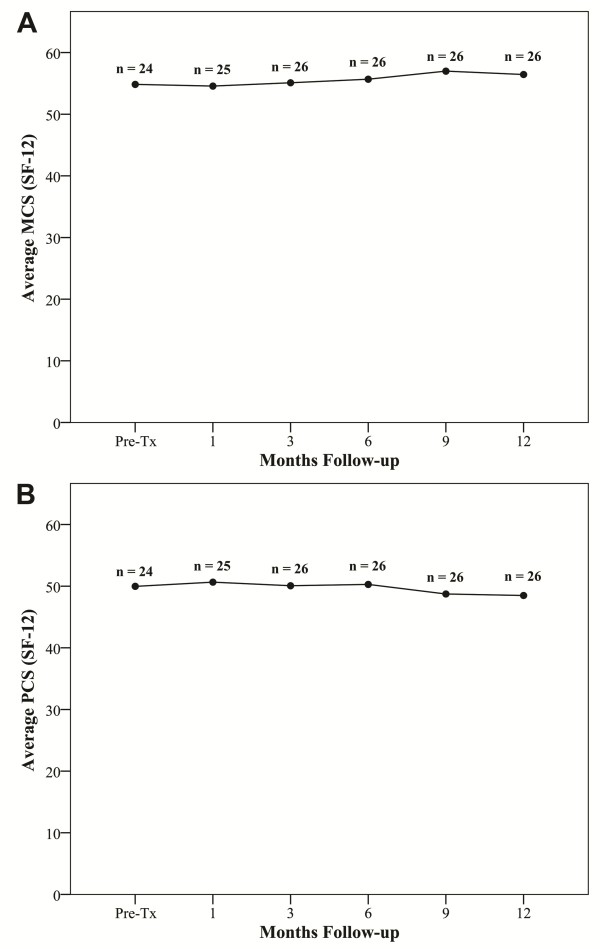
**Short Form-12 (SF-12) Health Survey quality of life: (A) SF-12 physical component score (PCS) and (B) SF-12 mental component score (MCS)**. The graphs show unadjusted changes in average scores over time. The scores range from 0 - 100 with higher values representing improved health status. Numbers above each time point indicate the number of observations contributing to the average.

**Figure 5:**
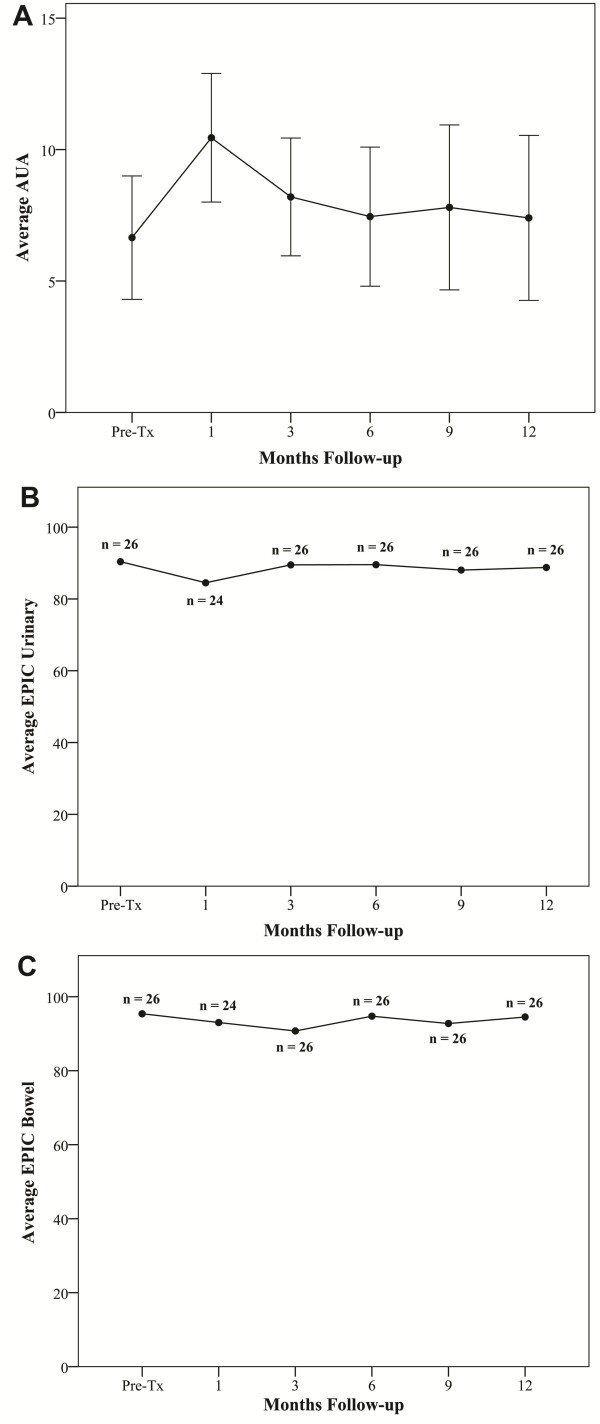
**Urinary and bowel quality of life using the American Urology Association (AUA) score and the Expanded Prostate Cancer Index Composite (EPIC): (A) AUA score, (B) EPIC urinary and (C) EPIC bowel**. The graphs show unadjusted changes in average scores over time for each domain. AUA scores range from 0 - 35 with higher values representing worsening urinary symptoms. EPIC scores range from 0 - 100 with higher values representing a more favorable health-related QOL. Numbers above each time point indicate the number of observations contributing to the average. Error bars indicate 95% confidence intervals.

Toxicity has been minimal with no Grade 3 or higher gastrointestinal (GI) or gastrourinary (GU) toxicities (Table 2). Grade 1 and 2 acute toxicities included urinary symptoms requiring alpha blockers and bowel frequency/spasms requiring antidiarrheals. At one year post-treatment, the patients' perceptions of their physical (Figure 4A) and mental health (Figure 4B) were unchanged (Table 3). At one month post-treatment the mean AUA toxicity increased to 10.8 from a baseline of 6.8 (*p *= 0.0001), and the mean EPIC urinary score decreased to 82.7 from a baseline 90.5 (*p *= 0.0001), see Figures 5A and 5B and Table 3. Both mean AUA and EPIC urinary scores returned to baseline by one year after treatment. At one month post-treatment, the mean EPIC bowel score declined to 91.7 from a baseline of 95.7 (*p *= 0.042) (see Figure 5C and Table 3) and returned to baseline by one year after treatment.

**Table 2:** Summary of CTC graded acute gastrointestinal (GI) and genitourinary (GU) toxicities

		Pre-Tx		1 Month		3 Month		6 Month	
Gastrointestinal									
Toxicity	Grade	N	%	N	%	N	%	N	%
Diarrhea	0	21	(81)	19	(73)	21	(81)	20	(77)
	1	5	(19)	7	(27)	5	(19)	6	(23)
	2	0	(0)	0	(0)	0	(0)	0	(0)

Proctitis	0	26	(100)	22	(85)	23	(88)	24	(92)
	1	0	(0)	4	(15)	3	(12)	2	(8)
	2	0	(0)	0	(0)	0	(0)	0	(0)

Rectal	0	25	(96)	25	(96)	25	(96)	25	(96)
Bleeding	1	1	(4)	1	(4)	1	(4)	1	(4)
	2	0	(0)	0	(0)	0	(0)	0	(0)

Highest GI	0	20	(77)	16	(62)	19	(73)	19	(73)
	1	6	(23)	10	(38)	7	(27)	7	(27)

		Pre-Tx		1 Month		3 Month		6 Month	
Genitourinary									
Toxicity	Grade	N	%	N	%	N	%	N	%

Hematuria	0	26	(100)	26	(100)	26	(100)	26	(100)
	1	0	(0)	0	(0)	0	(0)	0	(0)
	2	0	(0)	0	(0)	0	(0)	0	(0)

Dysuria	0	22	(85)	17	(65)	25	(96)	25	(96)
	1	4	(15)	9	(35)	1	(4)	1	(4)
	2	0	(0)	0	(0)	0	(0)	0	(0)

Incontinence	0	25	(96)	18	(69)	19	(73)	21	(81)
	1	1	(4)	7	(27)	7	(27)	5	(19)
	2	0	(0)	1	(4)	0	(0)	0	(0)

Urinary	0	26	(100)	23	(88)	23	(88)	23	(88)
Freq/Urg	1	0	(0)	3	(12)	3	(12)	3	(12)
	2	0	(0)	0	(0)	0	(0)	0	(0)

Retention	0	14	(54)	5	(19)	9	(35)	8	(31)
	1	12	(46)	14	(54)	10	(38)	12	(46)
	2	0	(0)	7	(27)	7	(27)	6	(23)

Highest GU	0	13	(50)	5	(19)	7	(27)	8	(31)
	1	13	(50)	14	(54)	12	(46)	12	(46)
	2	0	(0)	7	(27)	7	(27)	6	(23)

	2	0	(0)	0	(0)	0	(0)	0	(0)

**Table 3:** Overview of patient quality of life (QoL)

	Pre-Treatment	1 Month	3 Month	6 Month	9 Month	12 Month
SF-12 PCS	50 (35.2 - 58.9)	50.9 (31.4 - 61.4)	50.5 (31.4 - 61.2)	50.6 (25.7 - 56.7)	49 (27.1 - 57.2)	49 (27.6 - 59.8)
SF-12 MCS	54.8 (37.2 - 61.3)	54.4 (41.2 - 61)	55.2 (37.3 - 63.2)	55.7 (34.5 - 61.5)	**57 (47.1 - 64.7)**	56.5 (38.5 - 62.6)
AUA	6.8 (1 - 21)	**10.8 (3 - 20)**	**8.1 (1 - 21)**	7.7 (1 - 23)	7.5 (2 - 26)	7.4 (0 - 22)
SHIM	17.2 (3 - 25)	16 (1 - 25)	15 (1 - 25)	15.2 (1 - 25)	15.6 (1 - 25)	14.3 (1 - 25)
EPIC Urinary	90.5 (63 - 100)	**82.7 (61.1 - 100)**	87.7 (53.7 - 100)	88.5 (65.8 - 100)	88.1 (68.6 - 100)	89 (60.2 - 100)
EPIC Bowel	95.7 (66.7 - 100)	**91.7 (62.5 - 100)**	92.6 (66.7 - 100)	94.1 (70.8 - 100)	94.1 (62.5 - 100)	94.8 (75 - 100)
EPIC Sexual	66.7 (27.8 - 95.8)	66.4 (20.8 - 100)	59.9 (0 - 100)	**59.8 (0 - 100)**	**60 (16.7 - 100)**	60.1 (13.8 - 100)
EPIC Hormonal	94.2 (75 - 100)	**90.9 (70 - 100)**	90.8 (60 - 100)	92.3 (60 - 100)	93.6 (60 - 100)	92.1 (60 - 100)

The table shows unadjusted changes in mean toxicity and QOL scores over time. SF-12 scores range from 0 - 100 with higher values representing improved health status. AUA scores range from 0 - 35 with higher values representing worsening urinary symptoms. SHIM scores range from 0 - 25 with lower values representing worsening sexual function. EPIC scores range from 0 - 100 with higher values representing a more favorable health-related QOL. Bolded items signify a statistically significant change in reported QoL from baseline measured by Wilcoxon rank sum test at 0.05 significance level.

**Figure 6:**
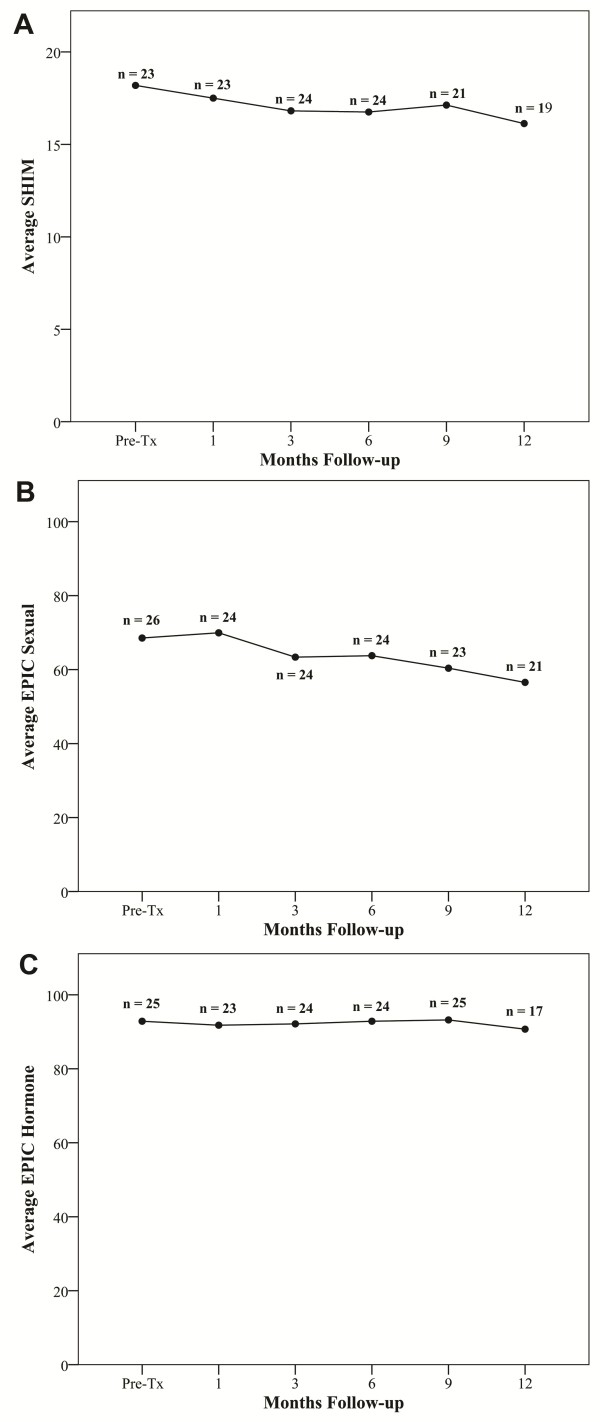
**Sexual quality of life using the Health Inventory for Men (SHIM) and Expanded Prostate Cancer Index Composite (EPIC): (A) SHIM, (B) EPIC sexual and (C) EPIC hormonal scores**. The graphs show unadjusted changes in average scores over time for each domain. SHIM scores range from 0 - 25 with lower values representing worsening sexual symptoms. EPIC scores range from 0 - 100 with higher values representing a more favorable health-related QOL. The graphs show unadjusted changes in average toxicity and QOL scores over time. Numbers above each time point indicate the number of observations contributing to the average.

Sexual dysfunction is a major criterion for the clinical diagnosis of hypogonadism [26]. At one year post-treatment, the mean SHIM decreased to 14.3 from a baseline of 17.2, and the mean EPIC sexual scores decreased to 60.1 from a baseline of 66.7 (Figures 6A and 6B, Table 3). However, these changes were small and not statistically (*p *= 0.126 and *p *= 0.341, respectively) or clinically significant [27]. At one month post-treatment, the mean EPIC hormone score declined to 90.9 from a baseline of 94.2 (*p *= 0.039); it returned to baseline by one year post-treatment (Figure 6C and Table 3).

## Discussion

Pelvic irradiation causes a dose-dependent reduction in serum testosterone levels that increases with larger field sizes and higher testicular doses [28]. For conventional pelvic radiation therapy, the drop is approximately 10-30%; this reaches a nadir, on average, several months post-treatment and can persist for years thereafter [28-33]. In addition to precipitating clinical hypogonadism, with its adverse effects [15], this testosterone decline may undermine the utility of PSA as a tumor response marker [10]. Radiation dose escalation, hypofractionation, and the increased total body radiation with multi-field treatments [34] and image guidance [35] could enhance this testosterone decline. Thus, this study was aimed to assess the risk of biochemical and clinical hypogonadism following CyberKnife SBRT monotherapy for clinically localized prostate cancer.

In our study, we observed a small decline (23.75%) in total testosterone levels after SBRT treatment consistent with that reported by others [36] and similar to that seen with conventional prostate radiation therapy [30]. This decline in testosterone was unlikely responsible for a promising 12-month PSA nadir as variations in serum testosterone do not greatly affect PSA levels in eugonadal men [37,38]. It remains to be determined whether testosterone decreases are temporary or permanent as these levels can take years to normalize [28]. Future studies will determine if testosterone levels fully recover to age-appropriate levels in our patient population.

The cause of this testosterone decline is unknown. Leydig cell dysfunction due to testicular scatter irradiation (mean dose of 2-4 Gy) in older men has been proposed as the major causative factor [12,29,31-33]. However, normal age-related testosterone decline [25] and treatment related stress [39] may also contribute. To determine if emotional and physiological stress could be responsible for our small decline in total testosterone, we examined acute toxicity and quality of life indicators. Acute Grade 2 GU and GI toxicities were observed in 27% and 0% of patients, respectively (Table 2). There were no Grade 3 or higher acute toxicities. These results appear comparable to other published external beam radiation therapy series [19,40,41]. In the opinion of the authors, it is unlikely that these minimal toxicities were responsible for the observed decline in serum testosterone. Consistent with findings of others, the small decline in total testosterone had minimal effects on quality of life [42]. Our AUA, SHIM and EPIC scores returned to baseline by one year after treatment (Table 3 and Figures 5 and 6). This is not unexpected as a total testosterone of 8 nmol/L is likely adequate for normal physiologic and sexual functioning [18]. Whatever the cause, the small decline in total testosterone does not appear to be clinically significant as it did not adversely affect the utility of the PSA as a measure of tumor response or induced clinical hypogonadism.

## Conclusions

Hypofractionated SBRT is a promising new treatment option for men with low- and intermediate-risk prostate cancer. Early results suggest encouraging biochemical response with low toxicity and a low rate of new biochemical and clinical hypogonadism one year after treatment Investigation of more patients with longer follow-up is required to validate these conclusions.

## List of abbreviations used

AUA: American Urological Association; CTC: Common Toxicity Criteria; CTV: clinical target volume; DVH: dose-volume histogram; EPIC: Expanded Prostate Cancer Index Composite; GI: gastrointestinal; GU: genitourinary; GTV: gross target volume; ISSAM: International Society for the Study of the Aging Male; NCI: National Cancer Institute; NPO: nothing by mouth; PTV: planning target volume; QoL: quality of life; SHIM: Sexual Health Inventory for Men; SF-12: Short Form-12; and SBRT: stereotactic body radiation therapy.

## Declaration of Competing interests

BT Collins serves as a clinical consultant to Accuray Inc.

The other authors declare that they have no competing interests.

## Authors' contributions

EO and SS participated in data collection, data analysis, manuscript drafting, table/figure creation and manuscript revision. HH, JK, BE, JR, NP, BS, DB, and VC participated in data collection, data analysis and manuscript revision. SL, XY and GZ participated in treatment planning, data collection, data analysis, and manuscript revision. GB, NC, SD, GB, JP, KM and JL participated in treatment planning, data analysis and manuscript revision. LA, RJ, ND, BC, and AD participated in the design and coordination of the study. SC drafted the manuscript, designed the study, and led the research effort. All authors have read and approved the final manuscript.
